# Kleinbetriebliche Wirtschaftsstruktur — ein regionaler Resilienzfaktor in der Corona-Krise?

**DOI:** 10.1007/s10273-021-2823-7

**Published:** 2021-01-19

**Authors:** Petrik Runst, Jörg Thomä, Katarzyna Haverkamp, Till Proeger

**Affiliations:** Volkswirt. Inst. f. Mittelstand u. Handwerk, Universität Göttingen, Heinrich-Düker-Weg 6, 37073 Göttingen, Deutschland

**Keywords:** R10, R12, E32, H00, J60

## Abstract

Regionalspezifische Wirtschaftsstrukturen haben einen Einfluss auf die Resilienz von Regionen in konjunkturellen Krisenzeiten. Noch unklar ist in diesem Zusammenhang die relative Bedeutung kleinerer Unternehmen. Haben sie hinsichtlich der Arbeitslosigkeitsentwicklung in der Corona-Pandemie als Stabilisator oder als Krisenverstärker gewirkt? Unsere Ergebnisse zeigen, dass ländliche und durch die Handwerkswirtschaft geprägte Regionen konjunkturell weniger von den negativen Arbeitsmarktfolgen der Krise betroffen waren. Als zentraler Befund zeigt sich, dass Kreise mit kleinbetrieblicheren Wirtschaftsstrukturen eine höhere Resilienz als Kreise mit großbetrieblicherer Struktur aufweisen.

## Abstract (en)

Regional economic patterns affect the resiliency of geographic regions with regards to business cycle shocks. In particular, the literature has not sufficiently investigated the role of smaller companies. Do they serve as stabilisers or enhancers of business cycle fluctuations? Our results show that rural regions, as well as regions with a larger share of small companies — e. g. crafts companies — exhibit less severe labour market impacts as a result of the ongoing coronavirus crisis. Overall, our research points to the higher degree of resilience in regions with small scale firm structure.

Die ökonomische Resilienz von Regionen in Krisenzeiten weist eine hohe Heterogenität auf (Martin, 2012). Das heißt, sowohl die durch eine Krise – wie aktuell durch die Corona-Pandemie – ausgelöste Veränderung konjunktureller Indikatoren (z. B. Arbeitslosigkeit und Wirtschaftswachstum) als auch deren Erholung zwischen regionalen Einheiten ist sehr unterschiedlich ausgeprägt. In diesem Zusammenhang wird in der Forschungsliteratur angenommen, dass regionalspezifische Wirtschaftsstrukturen (Branchenzusammensetzung, Exportorientierung, Grad der funktionalen Spezialisierung etc.) Einfluss auf die regionale Resilienz ausüben (Martin et al., 2016). Ein möglicher regionaler Resilienzfaktor, der bislang nur wenig Aufmerksamkeit erfahren hat, ist die Unternehmensgrößenstruktur, genauer gesagt die relative Bedeutung von kleineren Unternehmen für die regionale Entwicklung in Krisenzeiten im Vergleich zu anderen Betrachtungsregionen (Martin et al., 2016, 581).

**Dr. Petrik Runst**, **Dr. Jörg Thomä**, **Dr. Katarzyna Haverkamp** und **Dr. Till Proeger** sind wissenschaftliche Mitarbeiter und eine Mitarbeiterin am Volkswirtschaftlichen Institut für Mittelstand und Handwerk an der Universität Göttingen.

Untersuchungen des Instituts für Arbeitsmarkt- und Berufsforschung (IAB) lassen vermuten, dass kleinbetrieblich geprägte Wirtschaftsstrukturen in manchen Regionen Deutschlands zum coronabedingten Anstieg der Arbeitslosigkeit beitrugen. So zeigen Gürtzgen et al. (2020) auf Basis der IAB-Stellenerhebung, dass wegen der Corona-Krise in der ersten Jahreshälfte 2020 vor allem Kleinst- und Kleinbetriebe Entlassungen vorgenommen haben, vermutlich weil „gerade sie ein zu dünnes Finanzpolster [haben], um solche unvorhergesehenen Krisen ohne Beschäftigungsabbau zu überstehen“. Von der Krise betroffene mittlere und große Betriebe hätten dagegen häufiger Kurzarbeit angemeldet und auf Entlassungen verzichtet. Die Ergebnisse von Böhme et al. (2020) deuten in diesem Zusammenhang darauf hin, dass die Arbeitslosigkeit infolge der Corona-Krise in solchen Regionen stärker gestiegen ist, in denen innerhalb der sehr stark betroffenen Wirtschaftszweige zusätzlich ein hoher Beschäftigungsanteil in Kleinstbetrieben (1 bis 9 Beschäftigte) vorgelegen hat.

Waren kleinere Unternehmen folglich auf regionaler Ebene ein Verstärker der im Zuge der aktuellen Corona-Krise entstandenen wirtschaftlichen Verwerfungen? Dies würde im Widerspruch zu verschiedenen empirischen Arbeiten stehen, die den Zusammenhang zwischen Unternehmensgröße und Konjunkturentwicklung zum Gegenstand haben und – insbesondere mit Blick auf die Lage am Arbeitsmarkt – Hinweise auf eine konjunkturelle Stabilisatorfunktion kleinerer Unternehmen finden (Fendel und Frenkel, 1998; Davidsson et al., 1999; Varum und Rocha, 2013; Thomä, 2011). Vor diesem Hintergrund untersuchen wir in diesem Beitrag die Änderungen der Arbeitslosenquoten auf regionaler Ebene infolge der Corona-Pandemie unter besonderer Berücksichtigung der Unternehmensgrößenstruktur.

### Kleinere Unternehmen als konjunkturelle Stabilisatoren

Hinsichtlich der Absatzseite kann vermutet werden, dass kleinere Unternehmen aufgrund ihrer im Durchschnitt eher lokal-regionalen Geschäftsausrichtung weniger abhängig vom volatilen Exportgeschäft sind als größere Unternehmen. Außerdem können die komplexen und international verschränkten Produktionsvorgänge von größeren Unternehmen potenziell durch Lieferengpässe unterbrochen werden. Kleinere Unternehmen sind zwar ebenfalls von Vorprodukten abhängig, allerdings werden diese weniger stark international bezogen und können teilweise leichter über Lagerkapazitäten abgefedert werden. Zudem erfüllen kleinere Unternehmen oftmals eine Versorgungsfunktion für private und öffentliche Haushalte, wobei die dahinterstehenden Bedürfnisse auch in konjunkturellen Abschwüngen nicht oder nur in geringem Maße aufgeschoben werden. Aufgrund dieser Argumente wird kleineren Unternehmen insbesondere in Zeiten exportinduzierter Wirtschaftskrisen eine volkswirtschaftliche Stabilisatorfunktion zugeschrieben (Erixon, 2009; Thomä, 2012).

Mit Blick auf die Lage am Arbeitsmarkt wurde in früheren Studien eine konjunkturstabilisierende Wirkung kleinerer Unternehmen – insbesondere von solchen aus dem Handwerk – aus verschiedenen Gründen angenommen (Dürig et al., 2004; Erixon, 2009; Thomä, 2011; Thomä und Zimmermann, 2013). Aufgrund der geringeren Unternehmensgröße kommt für sie in konjunkturellen Krisenzeiten ein Beschäftigungsabbau als Bewältigungsstrategie per se weniger infrage als in Großunternehmen, denn die Entlassung eines Mitarbeiters reißt beispielsweise in einem Zehn-Personen-Betrieb relativ gesehen eine deutlich größere Lücke in die Personal- und Organisationsstruktur als in einem 250-Personen-Betrieb. Des Weiteren ist die Einstellung neuer Mitarbeiter:innen für Kleinst- und Kleinbetriebe mit relativ hohen Transaktionskosten (Bearbeitung von Bewerbungen, Einstellungsgespräche etc.) verbunden. Schließlich spielt personengebundenes Erfahrungswissen gerade in kleineren (Handwerks-)Unternehmen eine zentrale Rolle für die Innovations- und Wettbewerbsfähigkeit, sodass an Fachkräften auch in Krisenzeiten möglichst lange festgehalten wird, um schwer zu kompensierende Knowhow-Abflüsse infolge von Entlassungen zu vermeiden.

### Datensatz und deskriptive Ergebnisse

Die empirische Auswertung beruht auf Daten der Bundesagentur für Arbeit. Angaben zu Arbeitslosenquoten liegen monatlich und auf regionaler Ebene vor. Für alle Kreise wurden die Differenzen zum Vorjahresmonat gebildet und

Abbildung 1
**Veränderung der Arbeitslosigkeit**
Durchschnitt der Monate Juni, Juli und August 2019/2020

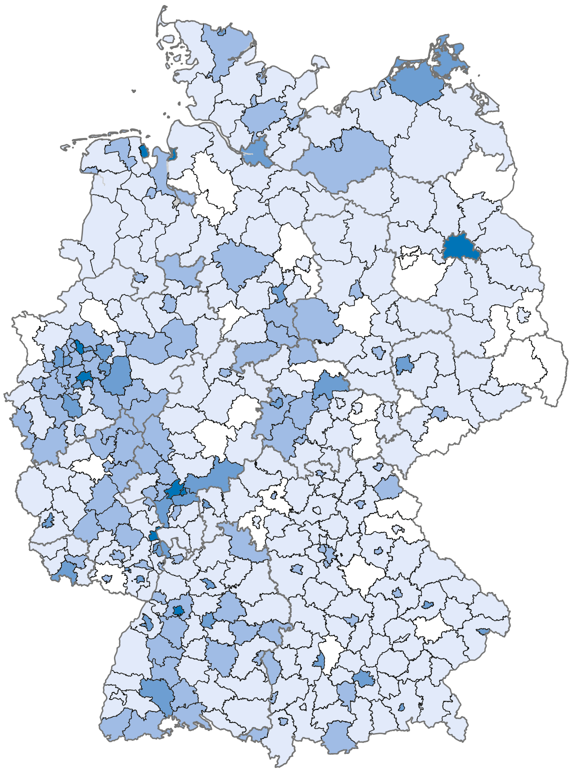

Anmerkungen: Dunklere Flächen stellen höhere Variablenwerte dar. Der Mittelwert der Änderung der Arbeitslosenquote beträgt 1,2 Prozentpunkte (Standardabweichung = 0,4). Der Minimal- und Maximalwert beträgt 0,23 % und 2,76 %.

Quelle: Bundesagentur für Arbeit, eigene Berechnungen.

in Abbildung 1 kartografisch abgetragen. Anhand der Abbildung lassen sich bereits erste Muster zur regionsabhängigen Betroffenheit von der Corona-Krise erkennen. Der Grad der Urbanisierung und die Wirtschaftsstruktur (z. B. Tourismus) stehen offenbar in einem Zusammenhang mit der Änderung der regionalen Arbeitslosenquoten. Hingegen zeigt der Vergleich mit Abbildung 2, dass der Zusammenhang mit der Corona-Betroffenheit (Fallzahlen pro 100.000 Einwohner:innen) nicht eindeutig ist. Ebenfalls bleibt offen, ob und inwiefern die Wirtschaftskraft einer Region im Zusammenhang mit der Änderung der Arbeitslosenquote steht.

Die Variablen der multivariaten Analyse und diesbezügliche deskriptive Statistiken werden in Tabelle 1 zusammenfassend dargestellt. Das Regionsmerkmal Brutto-

Abbildung 2
### Corona-Infektionszahlen pro 100.000 Einwohner:innen, Stand: Ende September 2020

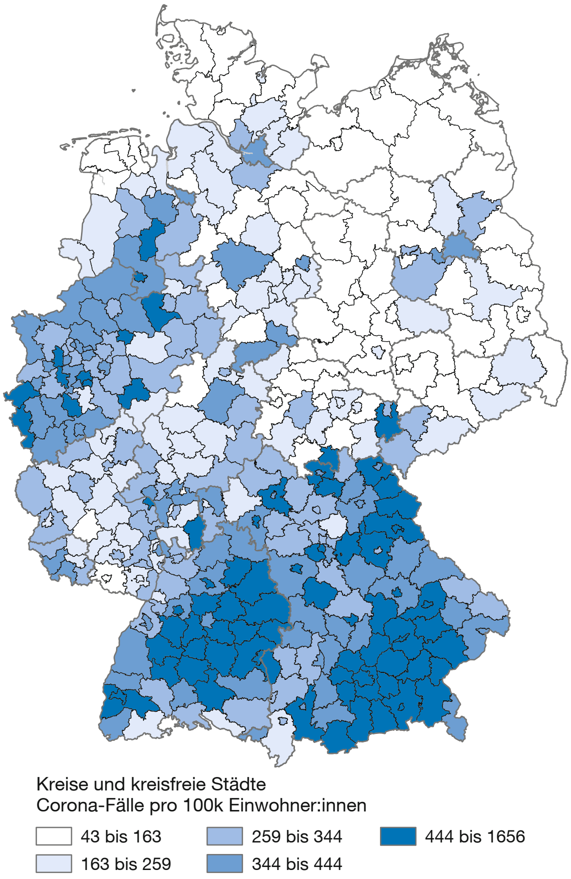

Anmerkungen: Dunklere Flächen stellen höhere Variablenwerte dar.

Der Minimal- und Maximalwert für die Infektionszahlen pro 100.000 Einwohner:innen betragen 42 und 1656.

Quelle: Robert-Koch-Institut, eigene Berechnungen.

Tabelle 1
### Variablenübersicht, deskriptive Statistik

| Variablen | Erläuterung | Mittelwert | St. Abw. |
|---|---|---|---|
| *Abhängig* | | | |
| Änderung der regionalen Arbeitslosenquote | Vergleich zum Durchschnitt der Vorjahresmonate Mai bis August | 1,207 | 0,407 |
| *Erklärend* | | | |
| Ländlicher Raum[a] | Dummy ländlicher Kreis | 0,506 | 0,500 |
| Ost | Dummy ostdeutscher Kreis | 0,192 | 0,394 |
| BIP/Kopf | Bruttoinlandsprodukt (BIP) pro Kopf in Euro | 37,094 | 16,054 |
| Starke Corona-Betroffenheit | Dummy: Infektionszahlen größer als Mittelwert | 0,426 | 0,495 |
| Wirtschaftliche Betroffenheit | Gewichteter Mittelwert (Σ Sektoranteil$_i$ mal Betroffenheitswert$_i$) | 2,541 | 0,137 |
| Handwerksregion | Dummy: Oberes Drittel der Verteilung (SV-Beschäftigte im Handwerk) | 0,329 | 0,471 |
| Kleinstbetriebe | 1-9 SV-Beschäftigte, Anteil an allen Betrieben in ‰ | 877,428 | 20,474 |
| Kleinbetriebe | 10-49 SV-Beschäftigte, Anteil an allen Betrieben in ‰ | 96,845 | 14,205 |
| Mittlere Betriebe | 50-249 SV-Beschäftigte, Anteil an allen Betrieben in ‰ | 22,083 | 6,113 |
| Großbetriebe | >250 SV-Beschäftigte, Anteil an allen Betrieben in ‰ | 3,644 | 1,504 |

Anmerkung: [a] Definition des Bundesinstitut für Bau-, Stadt-, und Raumforschung (BBSR) (www.raumbeobachtung.de/Raumabgrenzungen). Folgende Kriterien werden herangezogen: 1. Bevölkerungsanteil in Groß- und Mittelstädten; Einwohnerdichte der Kreisregion; 3. Einwohnerdichte der Kreisregion ohne Berücksichtigung der Groß- und Mittelstädte. Weiterhin nutzen wir den Anteil der sozialversicherungspflichtig Beschäftigten in allen Wirtschaftsabschnitten (A bis T).

Quellen: Bundesagentur für Arbeit; Bundesinstitut für Bau-, Stadt- und Raumforschung; Robert Koch-Institut.

inlandsprodukt (BIP) je Kopf, der Indikator für den ländlichen Raum, die Handwerksregionsvariable sowie die Angaben zur Unternehmensgrößenstruktur stammen aus der Regionaldatenbank INKAR des Bundesinstituts für Bau-, Stadt- und Raumforschung. Die Dummy-Variable „Handwerksregion" ist gleich eins, wenn sich der Kreis im oberen Drittel der Verteilung der sozialversicherungspflichtig Beschäftigten im Handwerk befindet. Sie liefert ein alternatives Maß für die „Kleinbetrieblichkeit" von Regionen. Denn gerade im Hinblick auf die Vielzahl der stark regional verankerten handwerklichen Kleinbetriebe wird in der Literatur von einem beharrenden Beschäftigungsverhalten im Konjunkturverlauf ausgegangen, das stärker ausgeprägt ist als in anderen kleinbetrieblich strukturierten Branchen (Dürig et al., 2004; Thomä, 2011). Entsprechend hoch wird die Bedeutung der Handwerkswirtschaft für die Stabilität von Regionen bewertet (Rudolph, 1997; Thomä, 2016). In diesem Zusammenhang ist wichtig, dass die Sicherung personengebundenen Erfahrungswissens über eine langfristige Mitarbeiterbindung gerade im Handwerk von hoher Bedeutung für die betriebliche Innovations- und Wettbewerbsfähigkeit ist (Thomä und Zimmermann, 2013, 2020). Schließlich nutzen wir die Variable „Corona-Infektionen pro 100.000 Einwohner:innen". Die hier zugrundeliegenden Daten wurden vom Robert-Koch-Institut bezogen.

Die Zusammensetzung der regionalen Wirtschaftsstruktur erscheint a priori von hoher Bedeutung für die Änderungen der Arbeitslosenquoten zu sein. Wir kontrollieren deshalb für den Anteil der sozialversicherungspflichtig Beschäftigten in allen Wirtschaftsabschnitten (A bis T),

Abbildung 3
**Deskriptiver Zusammenhang zur Unternehmensgröße**

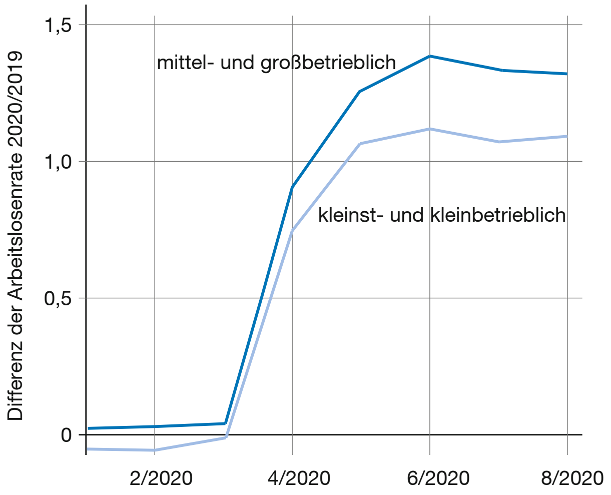

Anmerkung: Die beschreibende Variable differenziert Regionen danach, ob der Anteil der Kleinst- und Kleinunternehmen an allen Unternehmen über dem Durchschnitt aller Regionen liegt.

Quelle: eigene Berechnungen.

Abbildung 4
**Deskriptiver Zusammenhang zur Handwerksprägung einer Region**

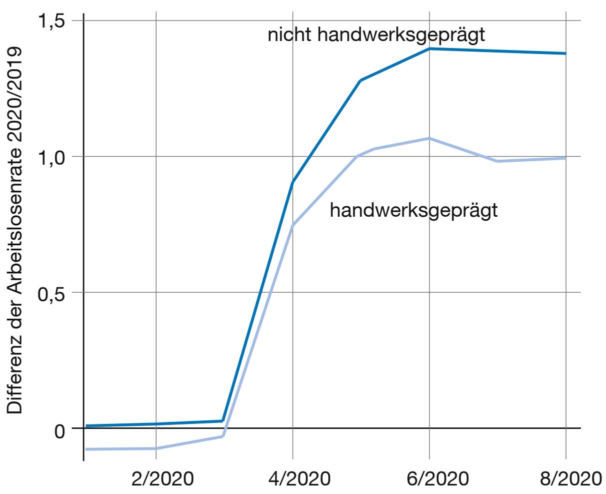

Anmerkung: Die beschreibende Variable differenziert Regionen danach, ob der Anteil der Handwerksbeschäftigten an allen SV-Beschäftigten im oberen Drittel der Verteilung über alle Regionen liegt.

Quelle: eigene Berechnungen.

die einem Sonderauszug der Bundesagentur für Arbeit entnommen wurden. In Bachtrögler et al. (2020) wurde für Österreich die Corona-Betroffenheit in den Abschnitten der Wirtschaftszweige abgeschätzt. Auf Grundlage dieser Studie generieren wir einen Betroffenheitsindex auf Kreisebene (gewichteter Mittelwert), in dem der Betroffenheitsindikator eines Wirtschaftsabschnitts i (auf einer Skala von 1 bis 5) mit dem jeweiligen Anteil der Beschäftigten im jeweiligen Sektor (Wirtschaftsabschnitte A bis T) multipliziert und anschließend über alle Wirtschaftssektoren A bis T aufsummiert wird.

Eine erste deskriptive Betrachtung der für unsere Fragestellung zentralen Variablen „Unternehmensgrößen-Verteilung“ und „Handwerksregion“ zeigt Folgendes (vgl. hierzu Abbildungen 3 und 4): Zu sehen ist die Entwicklung der regionalen Arbeitslosenquoten für die Monate Mai bis August 2020, jeweils bezogen auf den Vorjahresmonat. Regionen mit hohem Anteil an Kleinst- und Kleinbetrieben haben demnach im Betrachtungszeitraum einen geringeren Anstieg der Arbeitslosigkeit verzeichnet als Regionen mit einer eher mittel- und großbetrieblich geprägten Wirtschaftsstruktur. Ein ähnliches Muster zeigt sich bei der Betrachtung von Handwerksregionen. Entfällt ein hoher Anteil der SV-Beschäftigten einer Region auf die Handwerkswirtschaft, dann fällt der entsprechende Anstieg der regionalen Arbeitslosigkeit offenbar geringer aus als in weniger handwerklich dominierten Regionen von Deutschland.

## Regressionsergebnisse

Die Ergebnisse der OLS-Regression werden in Tabelle 2 dargestellt. Der Ländlichkeitsindikator steht in einem negativen Zusammenhang von moderater Stärke zur Änderung der Arbeitslosenquote, ebenso wie die Dummy-Variable für ostdeutsche Kreise. Im ländlichen Raum und in Ostdeutschland ist die Arbeitslosenquote weniger stark gestiegen als im Rest des Landes. Die Kreise, die überdurchschnittlich stark mit Corona-Infektionszahlen konfrontiert waren, weisen keine höheren Arbeitslosenquoten auf. Dies kann zum einen darauf hindeuten, dass das Infektionsgeschehen selbst offenbar keine direkte Auswirkung auf die Arbeitslosigkeit hat, der Angebotsschock in Form staatlicher Hygienemaßnahmen zur Eindämmung der Pandemie hingegen stärkere Auswirkungen hatte. Zum anderen zeigt es auch, dass die staatlichen Maßnahmen effektiv waren, indem die Infektionszahlen im internationalen Vergleich relativ gering blieben, sodass keine direkten Auswirkungen der Pandemie auf den Arbeitsmarkt beobachtbar sind. Der wirtschaftliche Betroffenheitsindex steht jedoch wie erwartet in einem starken positiven Zusammenhang zum Anstieg der regionalen Arbeitslosigkeit.

Die Kleinbetrieblichkeit einer Region (gemessen am Anteil der Kleinst- und Kleinunternehmen an allen Unternehmen) verringert die negativen Effekte auf die Arbeitslosenquote. Ein Anstieg um zwei Standardabweichungen im Falle des

Tabelle 2
**OLS Regression, abhängige Variable: Differenz der Arbeitslosenquoten mit dem Vorjahresmonat**

| | (1) | (2) | (3) | (4) |
|---|---|---|---|---|
| Ländlicher Raum | -0,229***<br>(0,000) | -0,203***<br>(0,000) | -0,155***<br>(0,000) | -0,193***<br>(0,000) |
| Ost | -0,103*<br>(0,091) | -0,115*<br>(0,054) | -0,239***<br>(0,001) | -0,0974*<br>(0,099) |
| BIP/Kopf | -0,000443<br>(0,815) | -0,000806<br>(0,660) | -0,00159<br>(0,463) | 0,000377<br>(0,828) |
| Starke Corona-Betroffenheit | -0,229***<br>(0,000) | 0,0460<br>(0,210) | 0,0691*<br>(0,059) | 0,0439<br>(0,230) |
| Wirtschaftliche Betroffenheit | | | | 0,402***<br>(0,004) |
| Unternehmens-Größenstruktur (Anteil in ‰) | | | | |
| Kleinstbetriebe | -0,0274***<br>(0,000) | -0,0251***<br>(0,000) | -0,0219***<br>(0,000) | -0,0236***<br>(0,000) |
| Kleinbetriebe | -0,0347***<br>(0,000) | -0,0317***<br>(0,000) | -0,0256***<br>(0,000) | -0,0290***<br>(0,000) |
| Mittlere Betriebe | Referenzkategorie | | | |
| Großbetriebe | 0,0181<br>(0,475) | 0,0124<br>(0,620) | 0,00839<br>(0,733) | 0,0135<br>(0,589) |
| Handwerksregion | | -0,124***<br>(0,001) | -0,0910**<br>(0,016) | -0,124***<br>(0,000) |
| Dummies Wirtschaftsabschnitt | nein | nein | ja | nein |
| _cons | 28,89***<br>(0,000) | 26,59***<br>(0,000) | 22,54***<br>(0,000) | 23,98***<br>(0,000) |
| N | 401 | 401 | 401 | 401 |

Anmerkung: p-Werte in Klammern, Residuen wurden nach Kreisen geclustered. * $p < 0{,}10$, ** $p < 0{,}05$, *** $p < 0{,}01$

Quelle: eigene Berechnungen.

Kleinunternehmensanteils (oder ca. 30 ‰) verringert den Anstieg der Arbeitslosenquote um ca. 0,8 Prozentpunkte. Bei einer entsprechenden Veränderung des Anteils der Kleinstunternehmen beträgt die Verringerung der Arbeitslosenquote ca. 1,4 Prozentpunkte. Des Weiteren bestätigt sich, dass die Handwerksprägung einer Region einen statistisch signifikanten Zusammenhang zur Entwicklung regionaler Arbeitslosigkeit hat. Handwerksregionen weisen einen geringeren Anstieg der Arbeitslosenquote in Höhe von 12,4 Prozentpunkten auf. Auffällig ist hier der relativ starke Zusammenhang. Dies dürfte unter anderem auch auf die Tatsache zurückzuführen sein, dass die Handwerkswirtschaft ausgehend von einer ausgesprochen positiven Konjunkturlage in die Corona-Krise geraten ist und sich gleichzeitig die Fachkräftesituation insbesondere im Handwerk kritisch darstellt. Entsprechend dämpfend wird daher ein hoher Beschäftigungsanteil im Handwerk auf die Entwicklung der regionalen Arbeitslosigkeit gewirkt haben.

## Fazit

Wir untersuchen die konjunkturelle Resilienz von deutschen Regionen anhand der Änderung der Arbeitslosenquoten zum Vorjahresmonat. Ostdeutsche und ländliche Kreise waren demnach am Arbeitsmarkt weniger stark von den negativen konjunkturellen Effekten der Corona-Krise betroffen. Zudem verringern eine kleinbetrieblich geprägte Wirtschaftsstruktur und die regionale Bedeutung der Handwerkswirtschaft den Anstieg der Arbeitslosenquote. Treten die Faktoren „Ländlicher Raum“, „Kleinbetrieblichkeit“ und „Handwerksregion“ in einer Region gemeinsam auf, dann zeigt sich auf Grundlage der Modellergebnisse kein Anstieg der regionalen Arbeitslosenquote infolge der Corona-Krise. Ein Zusammenhang mit den Infektionszahlen pro 100.000 Einwohner:innen sowie dem BIP je Kopf kann hingegen nicht festgestellt werden.

Es kann vermutet werden, dass die relativ geringere Exportabhängigkeit kleinerer Unternehmen sie vor dem nachfrageseitigen Schock (der auf den angebotsseitigen Schock folgte) geschützt hat. Weiterhin sind kleinere Unternehmen weniger stark in komplexe internationale Lieferketten eingebunden, die zum Teil unterbrochen wurden. Schließlich gehen wir davon aus, dass sich die Beschäftigungslage kleinerer Unternehmen in konjunkturellen Krisenzeiten im Durchschnitt stabiler darstellt als in größeren Betrieben. So tendieren Kleinst- und Kleinunternehmen beispielsweise aufgrund der höheren Bedeutung von personengebundenem Erfahrungswissen für den Erhalt der betrieblichen Innovations- und Wettbewerbsfähigkeit eher dazu, Mitarbeiter:innen in Krisenzeiten länger zu halten, unter anderem um in der Erholungsphase den Betrieb schneller aufnehmen zu können. Gerade in der kleinbetrieblich geprägten Handwerkswirtschaft ist dies der Fall.

Die in diesem Zusammenhang präsentierten empirischen Ergebnisse sprechen dafür, dass eine kleinbetriebliche Wirtschaftsstruktur tatsächlich ein regionaler Resilienzfaktor in Zeiten der Corona-Krise ist. Die Stabilisierungsfunktion kleiner (Handwerks-)Unternehmen wurde bereits im Zusammenhang mit der Wirtschafts- und Finanzkrise 2008/2009 beobachtet (Thomä, 2011). Abschließend stellt sich die Frage, ob die hier vorgestellten Ergebnisse im Widerspruch zur eingangs zitierten Studie von Böhme et al. (2020) stehen. Deren Ergebnisse deuten an, dass die Arbeitslosigkeit infolge der Corona-Krise in solchen Regionen stärker gestiegen ist, die eine kleinbetriebliche Struktur innerhalb der stark betroffenen Branchen aufweisen. Mit anderen Worten, wenn eine Branche besonders hart getroffen wurde, dann sind kleinere Unternehmen innerhalb dieser Branche vulnerabler als größere Unternehmen. Insgesamt gilt aber – dies zeigt die vorliegende Untersuchung –, dass Regionen mit einer kleinbetrieblichen Wirtschaftsstruktur (in mehr und weniger betroffenen Branchen) resilienter auf Krisen wie die aktuelle Corona-Pandemie reagieren als Regionen mit einem eher größerbetrieblichen Unternehmensbestand. Damit stehen die Ergebnisse der beiden Untersuchungen nicht im Widerspruch zueinander, sondern beleuchten stattdessen unterschiedliche Aspekte regionaler Resilienz.
